# Toxic Metal in Plant‐Based Therapeutics: Contamination Pathways, Oxidative Stress Induction, and Onset of Disease

**DOI:** 10.1155/tswj/7622430

**Published:** 2026-05-28

**Authors:** Nazmul Islam

**Affiliations:** ^1^ Department of Textile Engineering, Daffodil International University, Dhaka, Bangladesh, daffodilvarsity.edu.bd

**Keywords:** cellular damage, metal uptake, oxidative stress, plant-based therapeutics, toxic metals

## Abstract

Widespread environmental contamination by heavy metals from anthropogenic sources has raised significant concerns about their accumulation in plant based therapeutics and the associated risks to human health. With reported exceedances of permissible limits in approximately 20%–80% of plant based therapeutics across different geographical regions where over 30%–50% of the samples collected from high‐risk environments, particularly areas adjacent to industrial, mining, and intensive agricultural activities. The samples contain elevated concentrations of toxic metals, frequently exceeding World Health Organization (WHO) safety thresholds. This study elucidates the mechanisms through which toxic metals infiltrate plant‐based therapeutics and trigger oxidative stress, resulting in cellular dysfunction and disease progression. Under physiological conditions, reactive oxygen species (ROS) generated during normal metabolism are efficiently scavenged by endogenous antioxidants such as glutathione and superoxide dismutase, thereby preserving redox equilibrium. Exposure to redox‐active metals disrupts this balance by catalyzing cyclic reactions—such as Fenton chemistry—that result in excessive ROS generation. This elevated ROS production overwhelms endogenous antioxidant defenses, leading to oxidative stress characterized by lipid peroxidation, protein oxidation, and DNA damage. Such molecular damage triggers various cell death pathways, including apoptosis, necrosis, necroptosis, and ferroptosis. The contamination of herbal drugs by toxic metals occurs in two primary stages. During the herb development phase, plants absorb metals from contaminated soils. The efficiency of this uptake is quantified by the transfer coefficient (TC) and the translocation factor (TF), which indicate the accumulation of metals in the roots relative to the soil and the movement from roots to aerial parts, respectively. Soil metal availability is influenced by factors such as parent material, atmospheric deposition, agrochemicals, organic waste, and inorganic pollutants, while being modulated by processes like crop removal, leaching, and volatilization. In the manufacturing phase, the metal burden is further augmented by both intentional additions (e.g., metal bhasma with purported therapeutic benefits) and unintentional sources (e.g., post harvest processing, transportation, storage conditions, equipment interactions, and cross‐contamination). This comprehensive analysis underscores the urgent need for rigorous quality control measures throughout the herbal drug production process to mitigate metal‐induced oxidative stress and its deleterious health effects.

## 1. Introduction

Plant‐based therapeutics are herbal drugs that are prepared from different medicinal herbs [1, 2]. Affordability and availability fuel the popularity of plant‐based therapeutics, where the claimed safe and harmless advertisement of the medicines due to their natural origin plays a vital role [3, 4]. Plant‐based therapeutics are conventionally used for the treatment and prevention of different chronic and acute diseases [5–9]. However, there has been an increasing concern over the safety of these formulations due to their toxicity as the therapeutics are prone to contamination with metals due to their source and nature [10–13]. Herbs can be contaminated with metals during growing, harvesting, and processing. Metals are considered persistent pollutants due to their nonbiodegradability in nature [14–17]. Different natural and anthropogenic activities render them available in nature [18–21]. Continuous urbanization and industrialization in the developing world have led to high levels of metal contamination in the soil and surrounding environment [22, 23]. Entry routes for toxic metals in herbs in different stages are believed to be the following: polluted soils, industrial emissions, transportation, water used in irrigation, fertilizers and pesticides, and storage processes [5, 7, 8]. The manufacturing of herbs into finished formulations also contributes to metal contamination [3, 4].

Although certain elements like zinc (Zn), copper (Cu), manganese (Mn), and iron (Fe) are essential for biological functions, they can become toxic when present in high concentrations [16]. Conversely, elements such as mercury (Hg), lead (Pb), arsenic (As), hexavalent chromium (Cr^6+^), and cadmium (Cd) are classified as nonessential due to their toxicity, posing serious health risks even at very low levels [24–29]. Research conducted in various countries has indicated that herbal preparations often contain heavy metals exceeding the maximum permissible limits set by the World Health Organization (WHO) [24–30]. The interaction of metal ions with cellular components [31–34], including structural proteins, enzymes, and membrane systems, increases susceptibility to metal toxicity [35, 36]. Consequently, consumers face a significant risk of multiorgan damage [37] which may contribute to health conditions such as weakened immune responses, fetal abnormalities, cardiac disorders [38, 39], gastrointestinal cancers [37, 40, 41] and neurological [42, 43] and psychosocial impairments [44–46].

Ensuring the safety and quality of the therapeutics has become an increasing concern for the public health, regulatory authorities, and the pharmaceutical industry. Although numerous studies have documented the presence of toxic metals in the therapeutics, a critical research gap persists between the current pharmacokinetic understanding of these contaminants, particularly their absorption, translocation, and short‐term biological interactions, and their long‐term clinical outcomes in exposed populations. Current evidence primarily focuses on exposure levels and possible outcomes, whereas the downstream progression from bioaccumulation to chronic disease manifestation remains insufficiently documented. Moreover, existing regulatory frameworks often rely on the concentration limits without adequately weighing mechanistic toxicity data for cumulative and synergistic exposure effects across time. Consequently, uncertainties remain on how prolonged, low‐dose exposure translates into sustained oxidative stress, genomic instability, epigenetic alterations, and eventual disease onset. This research gap between exposure and clinical pathology highlights a important research area in understanding the full toxicodynamic continuum of toxic metals in plant‐based therapeutics.

Therefore, this study is aimed at minimizing the gap by critically evaluating recent advances in metal toxicology, where a precise emphasis on mechanistic pathways linking exposure to DNA damage, redox imbalance, cellular dysfunction, and disease progression has been given. Additionally, the review describes how toxic metals modulate gene expression and contribute to complex environmental and biological interaction networks, ultimately advancing a more integrated understanding of exposure‐to‐disease pathways.

## 2. Methodology

### 2.1. Publication Search

Three major bibliographic databases—Web of Science, PubMed, and Google Scholar—were selected for this study. A systematic literature search was conducted in both databases to collect relevant information. This review employed a systematic literature‐based approach to synthesize current evidence on metal‐induced toxicity, oxidative stress, onset of diseases, and possible contamination pathways in plant‐based therapeutics. Relevant peer‐reviewed articles, and reviews were retrieved from major scientific databases including Scopus, Web of Science, and PubMed, using targeted keywords such as “toxic metals,” “herbal medicine contamination,” “oxidative stress,” “reactive oxygen species (ROS),” and “metal‐induced toxicity.” Studies were screened for relevance, methodological rigor, and recency, prioritizing works published from 2005 to 2025. To enhance the comprehensiveness of the search, reference lists of the initially retrieved articles were also reviewed. Theses, dissertations, and conference proceedings were excluded to minimize potential bias. Emphasis was placed on research elucidating (i) the mechanisms of metal uptake and translocation in medicinal plants, (ii) the biochemical pathways of oxidative stress induction, and (iii) the health implications of metal‐contaminated herbal products. Comparative analyses were conducted to identify key patterns, mechanistic overlaps, and gaps in existing knowledge. The synthesized findings were critically evaluated to construct an integrated framework linking environmental contamination, phytochemical absorption, and cellular oxidative outcomes.

### 2.2. Data Extraction

**Figure 1:**
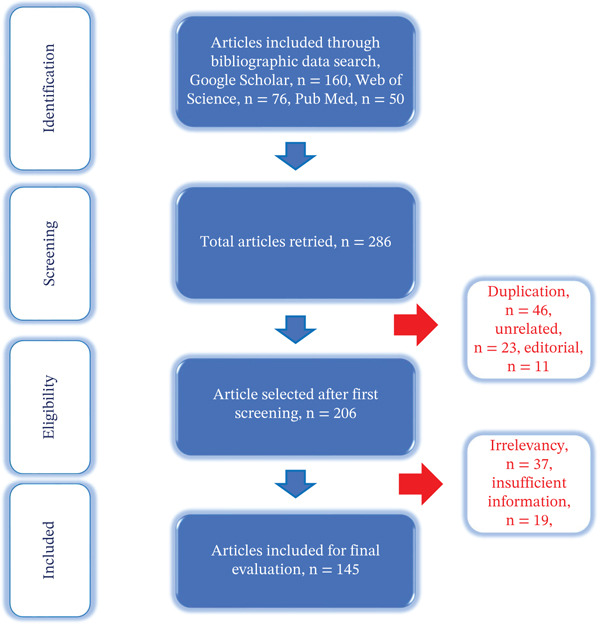
PRISMA flow diagram illustrating the systematic article selection process for identifying studies on mechanisms of metal‐induced toxicity.

All data from the finally selected studies were independently collected following a standardized protocol based on the Preferred Reporting Items for Systematic Reviews and Meta‐Analyses (PRISMA) guidelines. This approach was adopted to ensure transparency in presenting the rationale for the review, as well as to clearly document the authors′ efforts and the resulting findings. Each article was carefully read in full, and relevant information was manually extracted. The research synthesis process, outlining the text word–based article search strategy, is illustrated in Figure 1.

## 3. Findings

### 3.1. Pathway of Toxic Metals From Source to Plant‐Based Therapeutics

The environmental pathway of toxic metals involves their origin, movement, and eventual accumulation in herbal formulations, posing potential health risks [5, 8, 47]. This pathway can be broken down into key stages: sources of toxic metals and transfer of toxic metal from source to herbal formulation [3, 4]. Toxic metals originate from industrial activities [48, 49], agriculture, atmospheric deposition, and geological processes, contaminating soil, water, and air [50, 51], ultimately affecting ecosystems and human health [52–54]. Once released, toxic metals transfer from their sources to herbal formulations through various mechanisms. Soil contamination occurs as metals accumulate in the soil, where medicinal plants absorb them through their roots, leading to bioaccumulation. Water contamination further facilitates metal uptake when polluted water sources are used for irrigation. Airborne deposition also contributes, as metal‐laden dust and aerosols settle on plant surfaces. Additionally, contamination can occur during processing and storage through contact with polluted equipment, packaging, or environmental exposure [3, 4].

#### 3.1.1. Occurrence of Toxic Metals

**Figure 2:**
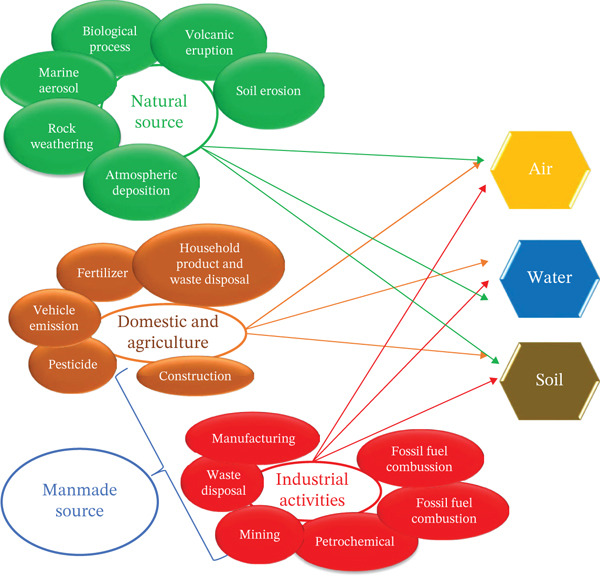
Natural and manmade sources of metals in the environment.

Heavy metal contamination arises from two primary pathways, namely geogenic [19–21] and anthropogenic sources [18, 55, 56] (Figure 2). Geogenic contamination is characterized by natural processes such as rock weathering, volcanic emissions, marine aerosol deposition, and biogeochemical cycling, which release and redistribute metals like Pb, As, Fe, Cu, and Zn into soils and water.

Anthropogenic contamination is mainly associated with human activities, including mining, smelting, fossil fuel combustion, industrial discharge, and agricultural practices. These activities add elevated concentrations of toxic metals such as Cd, Pb, Hg, and As into the environment, often creating localized hotspots with strong spatial variability. Field evidence consistently links industrial zones, urban areas, and intensively farmed lands with significantly higher metal accumulation compared with natural backgrounds [57–59]. Overall, geogenic sources define baseline metal levels, whereas anthropogenic activities are the dominant drivers of environmental enrichment and contamination severity.

Moreover, atmospheric deposition, both wet (via rainfall) and dry (via dust and aerosols), acts as a key transport mechanism, redistributing metals across vast geographic areas. Deforestation, soil erosion, and other land‐use changes exacerbate this issue by resuspending metal‐laden soils and sediments into surrounding ecosystems. In marine contexts, shipping activities, including fuel emissions and ballast water discharge, are significant contributors to coastal metal pollution.

Understanding the diversity and interconnectivity of these sources is essential for assessing ecological and human health risks associated with metal contamination, especially in sensitive sectors such as herbal drug production, where the accumulation of toxic metals can compromise both product safety and therapeutic efficacy [3–5, 7, 8].

#### 3.1.2. Transferring of Toxic Metals From Source to Plant‐Based Therapeutics

**Figure 3:**
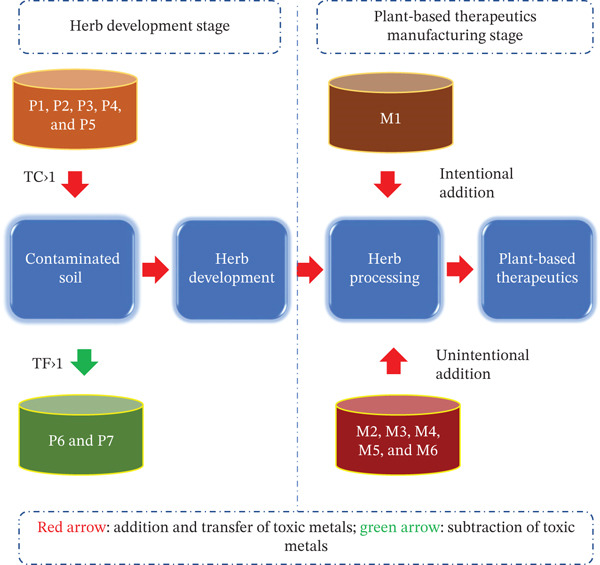
Schematic representation of pathway of toxic metals from source to plant‐based therapeutics.

From different sources, toxic metals find their way to plant‐based therapeutics in different steps. The transferring of toxic metals from the source to finished formulation is divided into two stages: development stage and manufacturing stage. Movement vector representing the transferring of metals is designated by arrow marks. In the herb development stage, herbs pick up toxic metals from contaminated soil. The availability of toxic metals in contaminated soil can be attributed to the following: P1: parent material, P2: atmospheric deposition, P3: agrochemical source, P4: organic waste, and P5: inorganic pollutants. However, the availability of toxic metals in soil is negatively affected by crop removal (P6) and losses by leaching and volatilization (P7) [3, 4]. The whole concept is represented in Figure 3.

The transfer coefficient (TC) and translocation factor (TF) are key parameters used to assess the uptake and movement of heavy metals in plants [60–62], including herbal drugs, which helps in evaluating contamination risks [63, 64]. The TC, also called the soil‐to‐plant transfer factor, measures the efficiency of metal uptake from soil to plant tissues (roots, stems, leaves, or medicinal parts) [65–67]. A higher TC indicates a stronger ability of the plant to absorb metals from the soil, whereas a lower TC suggests limited uptake. The TF assesses the movement of metals from roots to aerial parts (stems, leaves, or medicinal parts), indicating how metals are distributed within the plant. The TC and the TF are used to estimate the capacity to accumulate toxic metals from contaminated soils and to translocate them from the root to the stem, respectively [68]. The TC is designated as the ratio between the toxic metal concentration in the roots and its content in the soil. TC provides information about the ability of the herb to accumulate the toxic metals with respect to soil‐metal concentration. With TC value greater than 1 (8.7), the herb accumulates toxic metals, and TC value smaller than 1 indicates absence of metal uptake from contaminated soil [69–71]. TF indicates the ratio of the toxic metal concentration in the upper aerial part of the herb to that in roots, where its values higher than 1 (6.3) specify effective translocation of toxic metals from root to the aboveground plant part [72] (Table 1).

**Table 1:** A comparative perspective on metal uptake and internal distribution across different plant systems.

Study	Plant/matrix	Metal (s)	TC (soil → plant)	TF (root → shoot)	Key observation
Yoon et al. [71]	Native plants (contaminated site)	Pb, Cu, and Zn	> 1 for Zn, Cu in hyperaccumulators	> 1 in selected species	Efficient uptake and translocation in tolerant species
Olowoyo et al. [70]	*Jacaranda mimosifolia*	Multiple heavy metals	< 1–> 1 depending on metal	~1 or slightly > 1	Species‐dependent accumulation variability
Gajić et al. [69]	Phytoremediation plants	Various metals	Variable, up to > 1 in hyperaccumulators	Often > 1 in aerial parts	Strong phytoremediation potential in selected plants
Dinu et al. [72]	*Ocimum basilicum*	Cd, Pb, and Zn	> 1 in contaminated soils	> 1 for Cd and Zn	High translocation to leaves used medicinally
Adamczyk‐Szabela et al. [68]	*O. basilicum*	Heavy metals (thiuram exposure)	< 1–> 1 depending on exposure	Variable TF (> 1 under stress)	Stress‐induced enhanced translocation
General interpretation	—	—	TC > 1: active accumulation; TC < 1: limited uptake	TF > 1: efficient shoot translocation	Used for risk classification in herbal plants

Later, during the manufacturing stage, intentional and unintentional addition of toxic metals add up to the excess presence of toxic metals in finished formulations. Intentional addition includes metal bhasma (M1) assuming therapeutic benefit. Unintentional addition includes postharvesting treatments (M2), transportation in open bedded truck (M2), raw material storage in open places (M3), interaction with the container‐closure system (M4), interaction with processing equipment (M5), and cross contamination (M6).

#### 3.1.3. Source Identification

From different substrates, pollution sources can be identified. For this purpose, statistical tools are used for detecting metal contamination pathways that include various techniques for analyzing spatial distribution, and pollution source. Some commonly used methods include different multivariate analysis like principal component analysis (PCA), cluster analysis (CA), and correlation analysis (CoA), and Monte Carlo simulation which identify patterns and correlations among different metal pollutants and potential sources [32, 73–76].

In plant based therapeutic toxicity studies, multivariate statistical techniques such as PCA and CA have been widely employed to identify heavy metal contamination sources and to interpret complex pollution datasets. These analytical tools enable the characterization of contamination patterns in plant‐based therapeutics by distinguishing between different environmental and anthropogenic inputs responsible for metal accumulation.

PCA has been extensively applied to reduce the dimensionality of large datasets without sacrificing the most significant variance associated with toxic metal distributions in plant‐based therapeutics [77–80]. By transforming correlated metal variables into independent principal components, PCA facilitates the identification of dominant contamination sources. Usually, components with eigenvalues greater than 1.0 are typically retained based on the Kaiser criterion, and those explaining at least 80% of total variance are considered representative of major contamination pathways [75, 81]. The loading plots of metals such as Pb, Cd, As, and Hg within these components further aid in linking specific contaminants to their probable origins in cultivation and processing environments.

Similarly, CA has been effectively used to group samples based on similarities in toxic metal concentrations, thereby enabling the grouping of samples with comparable contamination profiles [26–29, 81]. In toxicity assessments, hierarchical clustering analysis (HCA) and K‐means clustering are commonly applied to normalized datasets obtained from different analytical techniques. These tools allow the differentiation of samples influenced by industrial, agricultural, or environmental pollution sources, whereas also highlighting spatial and processing‐related variability in contamination levels [82–84]. When combined with PCA‐based grouping, CA enhances the robustness of source apportionment and improves the interpretation of complex metal interaction patterns in plant matrices, thereby supporting risk assessment and regulatory evaluation altogether [32].

Finally, Monte Carlo simulation is a robust tool for assessing uncertainty and variability in metal contamination and identifying pollution sources in herbal drugs [85, 86]. Using random sampling and probabilistic modeling [87], it incorporates key variables—soil, water, air quality, and plant parameters like TC and TF [73, 76]—with probability distributions (normal, log‐normal, uniform) to reflect natural variability [88, 89].

### 3.2. Metal Induced Toxicity

From pollution source, through plant‐based therapeutics, toxic metals enter human body. After entering human body, toxic metals induce toxicity through which is illustrated in different studies. [24–29]. Metal‐induced toxicity, also known as heavy metal poisoning, is a serious health and environmental issue resulting from exposure to toxic metals such as lead (Pb), mercury (Hg), arsenic (As), cadmium (Cd), and chromium (Cr). Although some metals, like iron, zinc, and copper, are essential for biological functions, excessive exposure to toxic metals disrupts cellular processes, leading to severe physiological and neurological disorders. Metals exert toxic effects primarily by generating oxidative stress, interfering with enzyme functions, and disrupting cellular metabolism. Metal toxicity has severe health effects, impacting multiple organ systems. Neurologically, lead exposure is linked to cognitive impairments, learning disabilities, and behavioral disorders, especially in children, whereas mercury, particularly in its organic form (methylmercury), affects the central nervous system, leading to memory loss, tremors, and developmental delays. Renal and hepatic damage are also significant concerns, as cadmium accumulates in the kidneys, causing renal failure, proteinuria, and electrolyte imbalances, whereas arsenic and chromium contribute to liver toxicity, fibrosis, and an increased risk of hepatic cancers. Additionally, several heavy metals, including arsenic, cadmium, and chromium, are classified as carcinogens due to their ability to promote DNA damage, inhibit repair mechanisms, and induce epigenetic modifications, thereby increasing cancer susceptibility. Cardiovascular and respiratory complications further exacerbate the risks, with chronic exposure to lead and arsenic being associated with hypertension, atherosclerosis, and heart disease, whereas inhaled metal particles from industrial pollution contribute to lung inflammation, fibrosis, and chronic obstructive pulmonary disease (COPD) (Table 2).

**Table 2:** Toxic metals, their associated adverse health effects, and the safety limits established by the World Health Organization (WHO).

Metal	Primary target organs/systems	Major health effects	References	WHO safety limit in plant‐based therapeutics (mg/Kg) [4]
Lead (Pb)	Nervous system and cardiovascular system	Cognitive impairments, learning disabilities, behavioral disorders, hypertension, atherosclerosis, and heart disease	[26–29]; [24]	10
Mercury (Hg)	Central nervous system	Memory loss, tremors, developmental delays, and neurotoxicity (especially from methylmercury exposure)	[26–29]; [25]	1
Arsenic (As)	Liver, cardiovascular system, and respiratory system	Hepatic toxicity, fibrosis, hepatic cancers, hypertension, atherosclerosis, heart disease, and carcinogenesis	[26–29]; [24]	10
Cadmium (Cd)	Kidneys and liver	Renal failure, proteinuria, electrolyte imbalance, liver toxicity, and carcinogenesis	[25]	0.02
Chromium (Cr)	Liver and respiratory system	Liver fibrosis, hepatic cancer, lung inflammation, fibrosis, chronic obstructive pulmonary disease (COPD), and carcinogenesis	[26–29]	0.05
Iron (Fe)	Liver and cardiovascular system	Oxidative stress–induced tissue damage, liver injury, increased cardiovascular risk	[26–29]	n/a
Zinc (Zn)	Gastrointestinal system and nervous system	Nausea, vomiting, and neurotoxicity at high exposure levels	[24]	50
Copper (Cu)	Liver and nervous system	Wilson′s disease, hepatic and neurological disorders	[25]	3

Below is the comprehensive table comparing experimental heavy metal concentrations in plant‐based therapeutics against established regulatory limits (e.g., WHO/FDA standards) to show the magnitude of the risk (Table 3).

**Table 3:** Experimental heavy metal concentrations in plant based therapeutics against established regulatory limits (e.g., WHO/FDA standards).

Toxic metal	Plant/herbal matrix	Experimental concentration (mg/kg)	Regulatory limit (mg/kg)	Standard source	Reference
Cr	*Bacopa monnieri* (leaf)	13.19 ± 0.05	2.0	WHO	[90]
Cr	*Withania somnifera* (stem)	4.93 ± 0.02	2.0	WHO	[90]
Pb	Mixed herbal samples	50.11	10	WHO	[58]
Cd	*Curcuma longa*	6.20	0.3	WHO	[58]
Hg	*Chrysanthemum indicum*	8.69	0.2–0.5	WHO/FDA	[58]
As	*Plantago asiatica*	14.53	1.0–5.0	WHO	[58]
Hg	Plant based therapeutics (Bangladesh)	403	0.2	WHO	[4]
Ni	Plant based therapeutics (Bangladesh)	93	10	WHO	[4]
Pb	Indian medicinal plants	2.64	10	WHO	[90]
Cd	Indian medicinal plants	0.04	0.3	WHO	[90]

#### 3.2.1. Mechanism of Metal‐Induced Toxicity

##### 3.2.1.1. Protein/Enzyme Suppression

**Figure 4:**
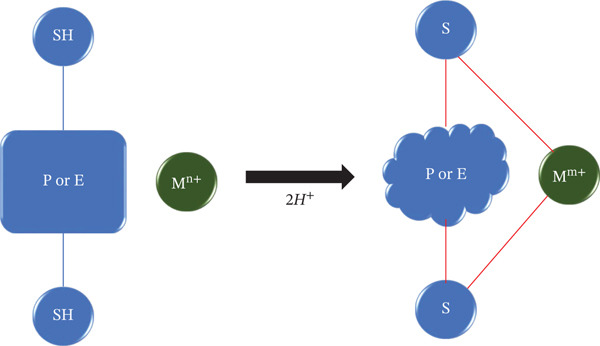
Mechanism of protein or enzyme suppression by toxic metals.

Metal exposure to humans occurs through environmental sources like pollution, contaminated food/water, or medical treatments. After entering the body, metal ions circulate in the bloodstream and can interact with biological molecules present in various tissues and cells [91]. Metal ions pose a high affinity for electron‐donating groups on proteins and enzymes. In vivo enzyme suppression of toxic metals is illustrated in Figure 4 where the active site of the protein/enzyme is represented by SH, the toxic metal is denoted by M with M^n+^ being its oxidation state, and M^m+^ is the reduced state [9].

Specific amino acid side chains (e.g., sulfhydryl groups in cysteine, imidazole groups in histidine, or carboxylate groups in aspartate/glutamate), and are prone to M^n+^ attack. As a result, binding to specific sites on proteins/enzymes take place. The binding can be reversible or irreversible, depending on the metal ion and the binding site, resulting in structural changes in proteins/enzymes. These changes may affect the protein′s active site geometry or overall stability. As a result, enhanced or inhibited enzymatic activity takes place due to the alteration of enzymatic activity and protein function. Cadmium has been demonstrated to inhibit human thiol transferases, including thioredoxin reductase, glutathione (GSH) reductase, and thioredoxin, in vitro by interacting with cysteine residues in their active sites, whereas methylmercury (MeHg) strongly suppresses the activity of l‐glutamine d‐fructose‐6‐phosphate amidotransferase in yeast [92, 93]. In some instances, the binding may result in the loss of normal function, impacting metabolic pathways or cellular signaling. The altered function of proteins and enzymes can disrupt normal cellular homeostasis and result in dysregulated cellular process. This dysregulation may contribute to oxidative stress, inflammation, or even cell death, ultimately impacting overall health. Through this process, metal ions cause toxicity to the human body by interacting with proteins and enzymes, resulting in the onset of diseases.

In plant, metal‐induced oxidative stress primarily disrupts the biosynthesis of plant secondary metabolites by interfering with redox homeostasis, enzymatic activity, and key metabolic pathways responsible for phenolics, flavonoids, alkaloids, and terpenoids production. Toxic metal stress can either upregulate or suppress secondary metabolite accumulation on the basis of exposure intensity, plant species, and duration of stress, with chronic exposure consistently linked with reduced biosynthetic efficiency and altered pharmacological potency of plant based therapeutics [35, 48, 94]. Mechanistically, toxic metals perturb central metabolic precursors derived from the shikimate, mevalonate, and phenylpropanoid pathways, thereby reducing the availability of key substrates required for flavonoid, phenolic, and alkaloid biosynthesis [95].

##### 3.2.1.2. Catalyzation of Redox Reactions

The mechanism of metal‐induced toxicity is primarily attributed to oxidative stress, a pathological condition arising from an imbalance between the generation of reactive oxygen species (ROS) and the cellular antioxidant defense systems. This imbalance disrupts redox homeostasis, leading to oxidative damage of vital biomolecules such as lipids, proteins, and nucleic acids, thereby promoting the progression of various diseases.

Under normal physiological conditions, cellular metabolism, particularly mitochondrial respiration, inevitably produces ROS as byproducts. However, these reactive species are tightly regulated by a complex network of enzymatic antioxidants and nonenzymatic antioxidants, which collectively maintain redox equilibrium. When cells are exposed to toxic metals, this regulatory balance is disrupted due to metals′ ability to either generate excess ROS or inhibit antioxidant enzymes, ultimately leading to oxidative injury and cellular dysfunction that underlie numerous pathological outcomes.

**Figure 5:**
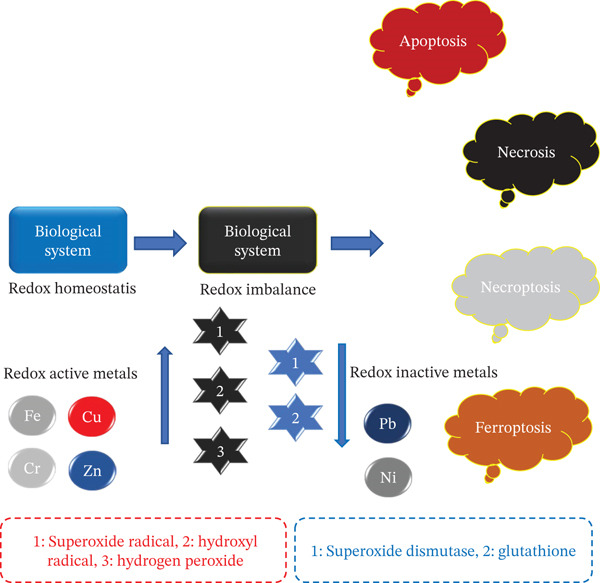
Schematic representation of metal induced oxidative stress and cellular damage.

Figure 5 provides a schematic illustration of the pathways through which metal exposure triggers oxidative stress, highlighting its underlying causes and biological consequences.

Molecules containing at least one oxygen atom and possessing unpaired electrons are known as ROS. These species include singlet hydroxyl, oxygen, and hydroperoxyl radicals [79, 80]. ROS are produced as a result of the incomplete breakdown of molecular oxygen, such as hydroxyl radicals (OH), hydrogen peroxide (H_2_O_2_), superoxide anion (O^2−^), and ozone (O_3_) [96]. Although ROS play a crucial role in various signaling pathways, they are generated within cells through a variety of physiological and biochemical processes. In aerobic organisms, these reactive species are vital for several biological functions. Pathway of generation of ROS includes superoxide radical, hydroxyl radical (mainly via Fenton reaction), and antioxidant enzyme includes GSH, superoxide dismutase (SDS) and so on. This condition represents cellular redox homeostasis [97–102]. The stability is hampered due to the action of toxic metals, which causes an imbalance between ROS production and guard.

Fenton and Fenton‐like reactions are highly significant because they serve as a key source of oxidative stress in all living organisms. The below reactions are examples of the Fenton reaction. Fenton′s reaction begins with the oxidation of ferrous ions (Fe^2+^) into ferric ions (Fe^3+^) in the presence of hydrogen peroxide (H_2_O_2_), which acts as an oxidizing agent. This process generates a hydroxyl radical (•OH) and a hydroxide ion (OH^−^) as byproducts. The reaction can be represented by the following equation:

(1) Fe2++H2O2⟶Fe3++OH−+•OH

In the subsequent stage, the ferric ion (Fe^3+^) is reduced back to the ferrous state (Fe^2+^) upon reacting with another hydrogen peroxide molecule. This step produces a hydroperoxyl radical (HOO•) along with a proton (H^+^), effectively regenerating the ferrous ion catalyst. The reaction is expressed as follows:

(2) Fe3++H2O2⟶Fe2++HOO•+H+

As a result, the overall Fenton process leads to the formation of two ROS, the hydroxyl radical (•OH) and the hydroperoxyl radical (HOO•). Additionally, hydroxide ions and protons formed during the process can combine to produce water. The net reaction can be summarized as follows:

(3) 2H2O2⟶•OH+HOO•+H2O

The overall result is the disproportionation of hydrogen peroxide, leading to the formation of two distinct oxygen‐radical species. These radicals, particularly the hydroxyl radical, are highly reactive and nonselective oxidizing agents. As Haber and Weiss proposed Reaction (1) as a component of what later became known as the Haber–Weiss reaction [103].

Probes into last few decades have shown that the redox active metals undergo redox cyclic reactions [104–106], generating reactive radicals like superoxide anion radical [95, 107, 108], nitric oxide, and so on. [109–111]. Therefore, redox homeostasis is disrupted and develops a condition called oxidative stress [48, 94, 112]. Oxidative stress is characterized by increased reactive oxygen nitrogen species production [113–115], shortage of intracellular antioxidant stores and free radical scavengers.

Metabolism and detoxification of ROS are also affected significantly due to enzymatic activity reduction/inhibition [116, 117]. In plant tissues, this disrupts the balance of the antioxidant defense system and directly impairs key enzymatic antioxidants. One of the earliest targets is superoxide dismutase (SOD), which normally converts superoxide radicals (O_2_•^−^) into H_2_O_2_. Excess ROS generated via the Fenton reaction overwhelms SOD activity, leading to enzyme inactivation through metal‐catalyzed oxidation of active site residues and disruption of metal cofactors required for catalytic function. Consequently, superoxide radicals accumulate, further fueling downstream oxidative reactions. Similarly, catalase (CAT), responsible for decomposing H_2_O_2_ into water and oxygen, becomes functionally compromised under sustained Fenton conditions. Raised intracellular H_2_O_2_ levels not only saturate CAT activity but also promote heme degradation within the enzyme, reducing its catalytic efficiency. This forms a feedback loop where excess H_2_O_2_ further increases hydroxyl radical generation through continued Fenton cycling. In parallel, peroxidases (PODs) and GSH peroxidase (GPx)‐like systems are severely affected. These enzymes depend on reducing equivalents such as GSH and NADPH to detoxify peroxides. However, Fenton‐resultant ROS oxidize GSH to GSSG, depleting cellular thiol pools and impairing enzyme regeneration. This leads to the fall of the ascorbate–GSH cycle, a core detoxification pathway in plant cells [104, 106, 118].

##### 3.2.1.3. Dysregulation of Gene Expression

The role of various signaling pathways in pathological processes caused by heavy metals has been reported [48, 117, 119]. Oxidative stress is a condition of biological system where increased ROS production overwhelms antioxidant protection and causes lipid peroxidation, protein modification, and DNA damage.

**Figure 6:**
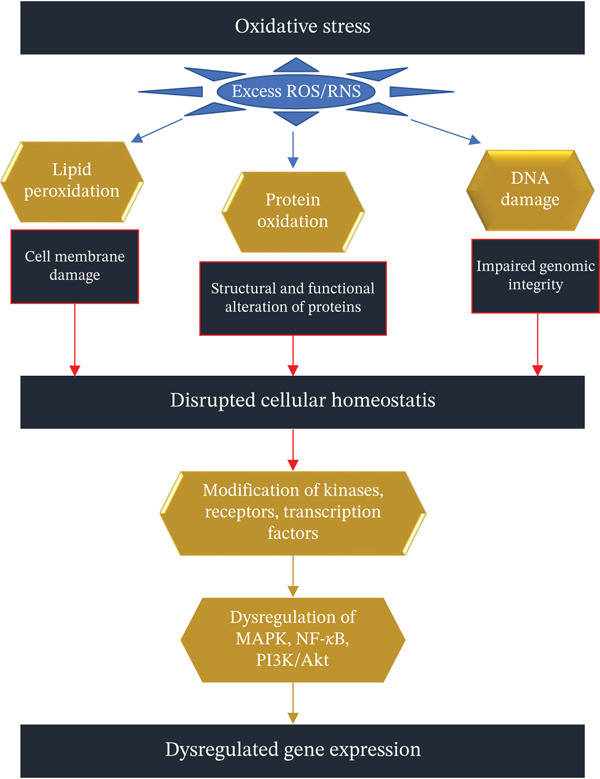
Dysregulation of gene expression due to the generation of oxidative stress.

In lipid peroxidation, membrane leakiness increases, leading to mutilation of membrane proteins, enzymes, and receptors [3, 104, 120]. Protein modification is characterized by the oxidation of sulfur‐containing side chains, chlorination of side chain amines, oxidation of histidines and tryptophans, and posttranslational modifications of proteins. DNA damage is due to the attack of ROS on the guanine present in DNA where guanine is oxidized by 8–8‐hydroxyguanine and 8‐hydroxy‐2‐deoxyguanosine, causing inappropriate protein formation. Depletion of the antioxidant enzyme GSH and bonding to sulfhydryl groups of proteins by the redox inactive metals are the primary causes of redox inactive metal‐induced toxicity [121, 122]. Cellular damage modifies critical signaling molecules like kinases, receptors, and transcription factors, altering signal transduction pathways such as MAPK, NF‐*κ*B, and PI3K/Akt. These disruptions contribute to dysregulated gene expression, further exacerbating cellular dysfunction and driving disease progression (Figure 6).

##### 3.2.1.4. Onset of Disease

Exposure to toxic metals and its subsequent oxidative stress severely disrupts key intracellular signaling networks, particularly the MAPK, NF‐*κ*B, and PI3K/Akt pathways. The pathways are central regulators of cell survival, inflammation; it begins with the generation of excessive ROS, which oxidatively inactivates regulatory phosphatases such as MAPK phosphatases and PTEN. As a result, sustained activation of MAPK and PI3K/Akt signaling take place where concurrently promote IKK complex activation and NF‐*κ*B nuclear translocation. This simultaneous activation creates a tightly interconnected signaling network. In the network stress perception is rapidly converted into persistent transcriptional reprogramming.

Precise activity between MAPK, NF‐*κ*B, and PI3K/Akt pathways amplifies and stabilizes pathological signaling outputs. ROS‐activated MAPK modules, particularly JNK and p38, enhance AP‐1–mediated transcription and synergize with NF‐*κ*B by increasing transcriptional coactivator recruitment. Consequently, intensifying expression of inflammatory and stress‐response genes [123] happens. In parallel, PI3K/Akt signaling potentiates NF‐*κ*B activation through IKK phosphorylation, additional reinforcing transcription of proinflammatory cytokines and antiapoptotic mediators. NF‐*κ*B reciprocally withstands oxidative stress by upregulating ROS‐generating enzymes, whereas MAPK‐driven transcriptional programs reinforce inflammatory priming. These two jointly develops a self‐amplifying feed‐forward loop. This reciprocal reinforcement safeguards pathway persistence even after the initial toxic exposure diminishes, thus locking cells into a chronic inflammatory and survival‐altered state.

The downstream consequence of this signaling crosstalk is a systemic shift from regulated homeostasis to persistent pathological remodeling which ultimately driving chronic disease progression. PI3K/Akt‐mediated inhibition of FOXO‐dependent transcription overwhelms antioxidant defenses and exacerbates ROS accumulation. On the other hand, NF‐*κ*B–driven antiapoptotic gene expression allows survival of genetically and metabolically damaged cells. This follow the disruption of the balance between apoptosis and regeneration [123]. These signaling abnormalities assimilate with documented metal‐specific toxic mechanisms, including manganese‐induced mitochondrial cytochrome c release and caspase cascade activation [124], nickel‐mediated epigenetic repression and HIF‐1*α* regulation [125], arsenic‐induced DNA damage and p53/p21 suppression [126, 127], mercury‐driven ROS‐mediated macromolecular injury [128], and lead‐induced disruption of zinc‐dependent transcriptional regulation [129]. Jointly, the convergence of MAPK–NF‐*κ*B–PI3K/Akt interaction delivers a mechanistic framework linking oxidative molecular injury to sustained inflammatory signaling, genomic instability, and the long‐term development of neurodegenerative disorders, cancer, cardiovascular disease, and metabolic dysfunction.

**Figure 7:**
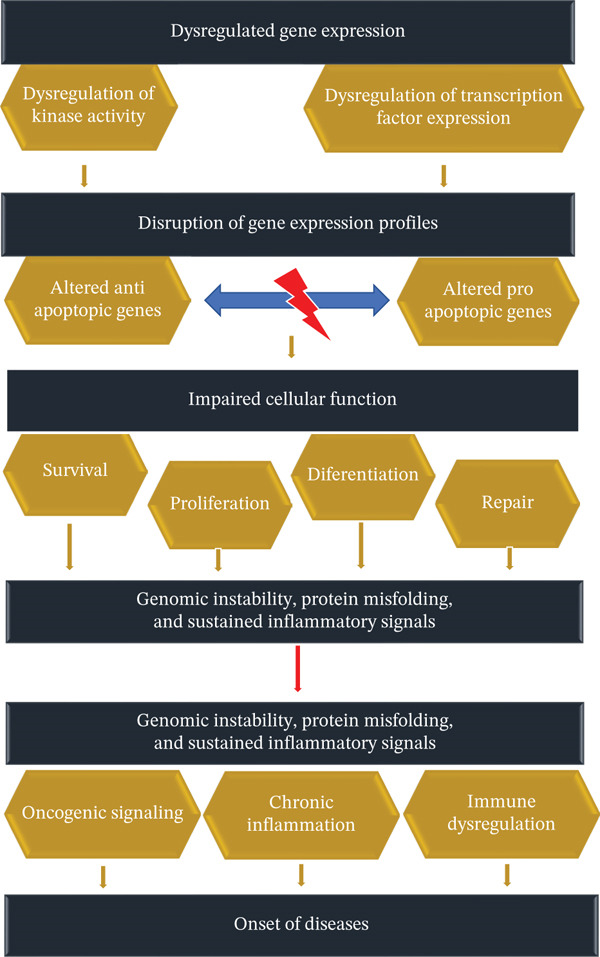
Onset of disease.

The onset of diseases caused by metal varies depending on the type of metal [130], level of exposure [91], duration, and individual susceptibility [131]. Acute toxicity occurs with high‐dose exposure over a short period, leading to immediate symptoms such as nausea, vomiting, abdominal pain, and neurological disturbances like confusion and seizures [132]. Chronic toxicity, which results from prolonged low‐dose exposure, has a more insidious onset, often manifesting after years or even decades [3, 4]. Symptoms may be vague initially, including fatigue, headaches, and mild cognitive decline, before progressing to severe neurological, renal, hepatic, cardiovascular, or carcinogenic effects. The delayed onset of chronic metal toxicity often makes diagnosis challenging, emphasizing the need for early detection and preventive measures (Figure 7).

### 3.3. Potential Effects of Toxic Metal Pollution on the Potency of Plant‐Based Therapeutics

Heavy metal pollution poses a significant threat to the potency and therapeutic efficacy of plant‐based medicines. The accumulation of toxic metals such as lead (Pb), cadmium (Cd), arsenic (As), mercury (Hg), and chromium (Cr) can alter the phytochemical composition of medicinal plants by disrupting key metabolic pathways involved in the biosynthesis of secondary metabolites, including alkaloids, flavonoids, phenolics, and terpenoids. These compounds are primarily responsible for the pharmacological activity of herbal preparations [47, 133, 134]. Metal‐induced oxidative stress damages cellular organelles and enzymes crucial for metabolite production, thereby reducing the concentration and bioavailability of active constituents. In some cases, metals may form complexes with phytochemicals, modifying their chemical structure, solubility, and bioactivity, which can lead to diminished therapeutic potential or even the emergence of toxic effects [135]. Furthermore, excessive ROS generation under metal stress can trigger premature senescence or inhibit plant growth [94, 136], leading to poor yield and compromised raw material quality. Consequently, heavy metal contamination not only undermines the pharmacological potency of herbal formulations but also raises serious safety and efficacy concerns for consumers.

## 4. Conclusions and Future Pathway

This study highlights the pathways through which toxic metal contamination occurs in herbal drugs and its role in inducing oxidative stress, ultimately leading to cellular damage and disease. Under normal physiological conditions, ROS are generated as byproducts of cellular metabolism and are effectively neutralized by antioxidant defense systems such as GSH and superoxide dismutase, thereby maintaining redox homeostasis. However, exposure to redox‐active metals disrupts this balance by catalyzing reactions, including Fenton chemistry, which leads to excessive ROS production. This oxidative stress results in biomolecular damage, including lipid peroxidation, protein oxidation, and DNA fragmentation, subsequently triggering various cell death pathways such as apoptosis, necrosis, necroptosis, and ferroptosis. Toxic metal contamination in herbal drugs occurs at two critical stages. During plant growth, metals are absorbed from contaminated soils, which is influenced by factors such as parent material composition, atmospheric deposition, agrochemical use, and industrial pollutants, whereas processes like crop removal, leaching, and volatilization further modulate metal availability. In the manufacturing phase, metal content increases due to both intentional additions (e.g., metal‐based preparations for therapeutic purposes) and unintentional contamination from processing, storage, transportation, and equipment interactions.

Considering the significant risks associated with toxic metal contamination in plant based therapeutics and its associated health risks, this study proposes a multitiered quality control framework to ensure product safety and therapeutic reliability. This framework stresses standardized testing of raw plant materials for toxic metals at the point of collection and entry into the production chain, alongside continuous and rigorous monitoring of the entire supply chain, from cultivation and harvesting to processing, packaging, and distribution. This type of integrated approach is crucial to prevent contamination at multiple entry points and to ensure traceability and accountability throughout the production process. By inserting systematic screening protocols and supply‐chain surveillance, the framework targets to minimize the risk of toxic metal exposure from the herbs and strengthen overall quality assurance in the plant based therapeutic production.

## Author Contributions

The author contributed to the study conception and design, data collection and analysis, draft preparation, editing and revision of the final draft.

## Funding

No funding was received for this manuscript.

## Conflicts of Interest

The author declares no conflicts of interest.

## Data Availability

Data sharing is not applicable to this article as no datasets were generated or analyzed during the current study.
